# The Onset of Parisi’s Complexity in a Mismatched Inference Problem

**DOI:** 10.3390/e26010042

**Published:** 2023-12-30

**Authors:** Francesco Camilli, Pierluigi Contucci, Emanuele Mingione

**Affiliations:** 1 The Abdus Salam International Center for Theoretical Physics, 34151 Trieste, Italy; fcamilli@ictp.it; 2 Dipartimento di Matematica, Alma Mater Studiorum—Universita di Bologna, 40127 Bologna, Italy; pierluigi.contucci@unibo.it

**Keywords:** statistical mechanics, spin glasses, high-dimensional inference, replica symmetry breaking

## Abstract

We show that a statistical mechanics model where both the Sherringhton–Kirkpatrick and Hopfield Hamiltonians appear, which is equivalent to a high-dimensional mismatched inference problem, is described by a replica symmetry-breaking Parisi solution.

## 1. Introduction

Beginning with Parisi’s seminal works on the Sherringhton–Kirkpatrick (SK) model [1,2], the ideas and the tools developed in spin glass theory spread across many other research fields such as computer science, probability theory, neural networks and others [3,4,5,6,7]. From a mathematical perspective, efforts to rigorously prove Parisi’s theory have yielded powerful techniques, such as interpolation methods [8,9], stochastic stability [10], and synchronization [11], which are currently instrumental in analyzing numerous disordered systems.

In this work, we will consider a family of mean-field spin glasses whose Hamiltonian contains two types of random interactions: the first is the SK type, while the second is a Hopfield model with a finite number of patterns. This class of models can also be interpreted as a high-dimensional inference problem known as a spiked Wigner model in a *mismatched* setting [12,13,14,15,16].

Our main result is a representation of the thermodynamic limit for the quenched pressure per particle in terms of a variational problem of Parisi type. The proof relies on two main ingredients: Guerra’s replica symmetry-breaking bound, which allows controlling the SK contribution, and adaptive interpolation, which is employed to linearize the Hopfield interaction. We start with a review of the SK model in Section 2 and then lay the ground for the inferential interpretation of the model under study in Section 3. In Section 4, we define and rigorously identify the exact solution of the model. Finally, we describe some interesting challenges for future investigations.

## 2. The SK Model

The SK model was introduced in the 1970s by D. Sherringhton and S. Kirkpatrick [17] and stands as an explicitly solvable mean-field spin glass. In their work, the authors discovered that the solution obtained through the replica symmetric (RS) approximation was not correct at low temperature. With a groundbreaking approach, Parisi identified a new type of solutions, nowadays called *replica symmetry breaking* (RSB), which proved to be correct at any temperature, thereby revealing a novel mathematical and physical structure [18].

The SK model is defined by its Hamiltonian, that is a function of *N* spins $\sigma ={\left(\sigma_{i}\right)}_{i\leq N}\in {\left\{-1,1\right\}}^{N}$:

(1) $$H_{N}^{SK}\left(\sigma \right)=-\frac{1}{\sqrt{2N}}\sum_{i,j\leq N}z_{ij}\;\sigma_{i}\sigma_{j}$$

where $z={\left(z_{ij}\right)}_{i,j\leq N}$ is a collection of i.i.d. standard Gaussian. In physical terms, the couplings between pairs of spins can be ferromagnetic or antiferromagnetic with equal probability. Consider also a random variable ξ with E|ξ|<∞ and a collection $\xi ={\left(\xi_{i}\right)}_{i\leq N}\overset{\mathrm{iid}}{∼}\xi$ representing random external fields acting on the spins. The *Parisi formula* is a representation for the large *N* limit of the pressure $p_{N}^{SK}$ defined by

(2) $$p_{N}^{SK}\left(\beta ,h\right)=\frac{1}{N}\log\sum_{\sigma \in {\left\{-1,1\right\}}^{N}}\exp\left(-\beta H_{N}^{SK}\left(\sigma \right)+h\;\sigma \cdot \xi \right)\;$$

In the definition (2), $\left(\beta ,h\right)\in R_{>0}\times R$ are fixed parameters, and the dependence on the realization of the random collections z,ξ is kept implicit. One can prove [5] that $p_{N}^{SK}$ converges, for almost all realizations of the disorder, to its average ${\bar{p}}_{N}^{SK}\left(\beta ,h\right)=Ep_{N}^{SK}\left(\beta ,h\right)$. Notice that E, taken after the logarithm, averages both the collections of z and ξ that are called *quenched variables*. The Hamiltonian (1) can also be regarded as a centered Gaussian process with covariance

(3) $$E\;H_{N}^{SK}\left(\sigma^{1}\right)\;H_{N}^{SK}\left(\sigma^{2}\right)=\frac{N}{2}q_{N}^{2}\left(\sigma^{1},\sigma^{2}\right)$$

where

(4) $$q_{N}\left(\sigma^{1},\sigma^{2}\right)=\frac{1}{N}\sum_{i=1}^{N}\sigma_{i}^{1}\sigma_{i}^{2}=\frac{1}{N}\;\sigma^{1}\cdot \sigma^{2}\;.$$

$q_{N}\left(\sigma^{1},\sigma^{2}\right)$ is the *overlap* between two spin configurations $\sigma^{1}$ and $\sigma^{2}$.

The Parisi variational principle for the limiting pressure per particle of this model was proved after almost three decades of efforts, and it is mainly due to the works of Guerra [8] and Talagrand [19]. We hereby summarize these milestones in a single theorem.

**Theorem** **1** (Parisi Formula [8,19]). *Let $M_{\left[0,1\right]}$ be the space of probability measures on [0,1], β>0 and y∈R. Consider the Parisi functional, which is defined as*

(5) $$\begin{array}{c} \chi \in M_{\left[0,1\right]}⟼P\left(\chi ;\beta ,y\right)=\log2+\Phi_{\chi }\left(0,y,\beta \right)-\frac{\beta^{2}}{2}\int_{0}^{1}dq\;q\chi \left(\left[0,q\right]\right)\;. \end{array}$$

*where $\Phi_{\chi }\left(s,y,\beta \right)$ solves the PDE*

(6) $$\begin{array}{c} \left\{\begin{array}{cc} \; & \partial_{s}\Phi_{\chi }=-\frac{\beta^{2}}{2}\left(\partial_{y}^{2}\Phi_{\chi }+\chi \left(\left[0,s\right]\right){\left(\partial_{y}\Phi_{\chi }\right)}^{2}\right)\\ \; & \Phi_{\chi }\left(1,y,\beta \right)=\log\cosh y\;. \end{array}\right. \end{array}$$

*The following holds*

(7) $$\lim_{N\to \infty }p_{N}^{SK}\left(\beta ,h\right)\;=\underset{\chi \in M_{\left[0,1\right]}}{\inf}E_{\xi }P\left(\chi ;\beta ,h\xi \right)\;a.s.$$

The key tool for the proof is the (Gaussian) *interpolation method*, which is introduced in [9] in order to prove the existence of the large *N* limit of ${\bar{p}}_{N}^{SK}$.

The thermodynamic equilibrium induced by the pressure ${\bar{p}}_{N}^{SK}$ is called *quenched equilibrium* and is defined as follows. Physical quantities (e.g., energy) are functions of the disorder variables z,ξ and the spin configurations σ. Given a function f(z,ξ,σ), its equilibrium value is defined as

(8) $$E{\langle f\rangle }_{N}:=E\sum_{\sigma \in {\left\{-1,1\right\}}^{N}}G_{N}\left(\sigma \right)f\left(z,\xi ,\sigma \right)$$

where $G_{N}$ is the (random) Boltzmann–Gibbs distribution

(9) $$\begin{array}{c} G_{N}\left(\sigma \right)=\frac{\exp\left(-\beta H_{N}^{SK}\left(\sigma \right)+h\;\sigma \cdot \xi \right)}{Z_{N}}\; \end{array}$$

The measure $E{\langle \;\;\rangle }_{N}$ is called a *quenched measure* and can be viewed as a two-step measuring process. Initially, for a given realization of the disorder variables z,ξ, one assumes that the system equilibrates according to the canonical Boltzmann–Gibbs distribution $G_{N}$ defining a (random) measure on the space of spin configurations. The expectation with respect to $G_{N}$ is denoted by ${\langle \;\rangle }_{N}$, namely

(10) $${\langle \;f\;\rangle }_{N}:=\sum_{\sigma \in {\left\{-1,1\right\}}^{N}}G_{N}\left(\sigma \right)f\left(z,\xi ,\sigma \right)$$

In probabilistic terms, $G_{N}$ defines a conditional measure given z and ξ. The remaining degrees of freedom z, ξ are then averaged according to their apriori distribution E.

An important role is played by the concept of *replicas*. Replicas are i.i.d. samples from $G_{N}$ at fixed disorder. Hence, the equilibrium value of a function $f(z,\xi ,\sigma^{1},\ldots ,\sigma^{n})$ of *n* replicas and the quenched variables z,ξ is defined by

(11) $$E{\langle f\rangle }_{N}=E\;\prod_{a\leq n}\;\sum_{\sigma^{a}\in {\left\{-1,1\right\}}^{N}}G_{N}\left(\sigma^{a}\right)f\left(z,\xi ,\sigma^{1},\ldots ,\sigma^{n}\right)\;.$$

The computation of derivatives of ${\bar{p}}_{N}^{SK}$ shows, using integration by parts, that the SK model is fully characterized by the (joint) distribution of the overlap array ${\left(q_{N}\left(\sigma^{l},\sigma^{l^{\prime }}\right)\right)}_{l,l^{\prime }\leq n}\equiv {\left(q_{l,l^{\prime }}\right)}_{l,l^{\prime }\leq n}$, namely the overlaps between any finite number *n* of replicas with respect to the measure (11). The main feature of the Parisi theory is the characterization of the mentioned joint measure by means of two structural properties:

(i) It is uniquely determined by a one-dimensional marginal, namely the distribution of $q_{1,2}$;
(ii) The distribution of three replicas has with a probability of one an *ultrametric* support
  (12) $$\lim_{N\to \infty }E\langle 1\left(q_{1,2}\geq \min\left(q_{1,3},q_{2,3}\right)\right)\rangle \;=\;1\;.$$

Despite having a mathematical proof of the Parisi Formula (7) for the SK model, (*i*) and (*ii*) have been rigorously proved only in the mixed *p*-spin model [6,20,21], an extension of the SK model, whose Hamiltonian contains also higher-order interactions (three-body, four-body, etc.).

One of the crucial instruments to achieve a rigorous control of the model is the so-called *Ruelle Probability Cascades* (RPCs), defined by Ruelle [22] when formalizing the properties of the Generalized Random Energy model of Derrida [23]. See also the characterization of RPC in terms of coalescent processes given in [24]. The first direct link between RPC and the SK model appeared in the work of Aizenman–Sims–Starr [25], where the authors found a representation of the thermodynamic limit of quenched pressure per particle in terms of the *cavity fields* distribution. This representation strongly suggested that if the thermodynamic limit of the overlap distribution is described by an RPC, then the Parisi formula is correct.

The first signal that the overlap array is described by an RPC was originally found by Aizenmann and Contucci in [10] with the identification of *stochastic stability* and by Ghirlanda and Guerra [26]. Both papers show an (infinite) set of identities for the moments of the overlap array distribution. It turns out that these identities actually imply that the support of the joint distribution of the overlap is ultrametric, as proved by Panchenko [27]. It should be noticed that Panchenko’s theorem requires identities for the overlap moments of all orders. The latter do not hold for the bare SK model, but it can be shown that there exists a perturbation of the Hamiltonian that forces the SK model to satisfy them without affecting the limit of the quenched pressure [28].

Once the validity of the Parisi Formula (7) is established, it is natural to ask for the properties of its solution. The uniqueness of the minimizer of (7) has been assessed by Auffinger and Chen [29], and its properties have been investigated for example in [30,31].

A relevant question about the minimizer is the following: for which values of the parameters (β,h) is the solution of (7) a Dirac-delta function $\delta_{q}$ for some q∈[0,1]? In this case, we say that the model is *replica symmetric* and the Parisi Formula (7) reads

(13) $$\lim_{N\to \infty }p_{N}^{SK}\left(\beta ,h\right)\;=\underset{q\in [0,1]}{\inf}\left\{\log2+E_{z,\xi }\log\cosh\left(\beta z\sqrt{q}+h\xi \right)+\frac{\beta^{2}}{4}{\left(1-q\right)}^{2}\right\}\;.$$

The replica symmetric region can be identified [6,32] with the region of parameters (β,h) where the overlap is a self-averaging quantity, namely

(14) $$\lim_{N\to \infty }E{\langle {\left(q_{1,2}-q^{*}\right)}^{2}\rangle }_{N}=0$$

where $q^{*}$ is exactly the value that realizes the infimum in (13). The physics conjecture is that the replica symmetric region can be identified by the so called Almeida–Thouless [33]

(15) $$\beta^{2}\;E_{z,\xi }\cosh^{-4}\left(\beta z\sqrt{q^{*}}+h\;\xi \right)\leq 1\;.$$

The above conjecture is proved only in the case of Gaussian external field $\xi_{i}∼N\left(0,1\right)$ [34]. An alternative characterization of the replica symmetric region has been obtained in [6,35]. If the minimizer corresponds to a non-trivial distribution (i.e., with non-zero variance), we say that *replica symmetry breaking* occurs, and the overlap is not a self-averaging quantity.

The Parisi formula has been extended to other mean field models with centered Gaussian interactions: vector spins [36], multispecies models [11,37,38], multiscale models [39,40]. Finally, we mention that the SK model fulfills a remarkable universality property: as long as $z_{ij}$’s are independent, centered, and with unit variance, the thermodynamic limit is still described by the Parisi solution [41].

In this work, we show that a class of non-centered Gaussian spin glasses admits an interpretation of high-dimensional inference that extends the celebrated correspondence between the spiked Wigner model and the SK model in the Nishimori line where replica symmetry is always fulfilled [3]. We show that the addition of an SK Hamiltonian to a Hopfield with a finite number of patterns can be mapped into a high-dimensional mismatched inference problem, where the statistician ignores the correct apriori distribution on the signal components they have to reconstruct. We shall see that even this slight mismatch may lead to the emergence of complexity, namely to the breakdown of replica symmetry, which is instead guaranteed under very mild hypotheses for *optimal* statisticians.

## 3. High-Dimensional Inference and Statistical Physics

High-dimensional inference aims at recovering a *ground truth* signal, ξ in the following, that is usually a vector with a very large number of components from some noisy observations of it, which is denoted by Y. The main feature of this setting is that the dimension of the signal, i.e., the number of real parameters to reconstruct, and the number of observations at disposal are a function of one another, typically a polynomial. For instance, for our purposes, ξ will be a vector of $R^{N}$ and Y will be an N×N matrix for a total of $N^{2}$ noisy observations. Hence, if the number of observations becomes large, the number of parameters to retrieve also does. Contrary to what happens in typical low-dimensional settings, where max-likelihood, or Maximum A Posteriori (MAP) approaches yield provably satisfactory reconstruction performances, in a high-dimensional setting, this is not always the case. In particular, one needs to devise another kind of more refined estimators that exploit the marginal posterior probabilities for each signal component.

Both approaches described above are Bayesian, and the knowledge of a prior distribution on the signal components can play a key role especially for high-dimensional problems. Furthermore, to compose the posterior measure for the entire signal, one needs the *likelihood* of the data, which is the probability of an outcome y of the variable Y *given* a certain ground truth realization ξ=x. As we shall discuss soon, under certain hypotheses, the Bayesian approach highlights the correspondence of relevant information theoretic quantities with thermodynamic ones. Among the others, a key quantity is the *mutual information* between the signal ξ and the observations Y, which quantifies the residual amount of information left in Y about ξ after the noise corruption. As intuition may suggest, the mutual information gives access to the best reconstruction error that is information theoretically achievable.

Finally, we stress that the high dimensionality of the problem can induce phase transition in some parameters of the model, like the so-called *signal-to-noise ratio* (SNR), that tunes the strength of the signal with respect to that of the noise in the observations.

### 3.1. Bayes-Optimality and Nishimori Identities

For the sake of simplicity, we start by considering a signal $\xi ={\left(\xi_{i}\right)}_{i\leq N}\in R^{N}$ of *i.i.d.* (independently and identically distributed) components $\xi_{i}\overset{\mathrm{iid}}{∼}P_{\xi }$, where $P_{\xi }$ has a finite fourth moment. The observations at the disposal of a statistician can be modeled as a stochastic function of the ground-truth signal: Y=F(ξ;z), where z is the source of randomness or simply the noise. Knowing the function F, from a Bayesian perspective, translates directly into having the likelihood of the model, namely the conditional distribution $dP_{Y|\xi =x}\left(y\right)=p_{Y|\xi =x}\left(y\right)dy$, which we assume to have a density $p_{Y|\xi =x}\left(y\right)$ over the Lebesgue measure. Observe that the likelihood is strongly affected by the nature of the noise.

According to Bayes’ rule, the posterior distribution of ξ given the data is:

(16) $$\begin{array}{c} dP_{\xi |Y=y}\left(x\right)=\frac{p_{Y|\xi =x}\left(y\right)dP_{\xi }\left(x\right)}{Z(y)}\;,\;Z\left(y\right)=\int p_{Y|\xi =x}\left(y\right)dP_{\xi }\left(x\right)\;, \end{array}$$

where $dP_{\xi }\left(x\right)=\prod_{i\leq N}dP_{\xi }\left(x_{i}\right)$, and Z(y) is the probability of a given realization of the data, which is sometimes also called *evidence*. In practice, the above posterior, which would be ideal to perform inference, is rarely available, and the statistician is not aware either of the likelihood or of the correct prior distribution for the signal, or even both. This motivates the following definition of a special inference setting:

**Definition** **1** (Bayes optimality). *The statistician is said to be Bayes optimal, or in the Bayes-optimal setting, if they are aware both of $P_{\xi }$ and F(·;z); namely, they have access to the posterior (16).*

The above is saying that an optimal statistician knows everything about the model except for the ground truth ξ itself. The Bayes-optimal setting is thus often used as a theoretical framework to establish the *information theoretical limits*. Indeed, it is known that the mean square error between the ground truth and an estimator $\hat{\xi }\left(y\right)$

(17) $$\begin{array}{c} \mathrm{MSE}\left(\hat{\xi }\right)=E{∥\xi -\hat{\xi }\left(y\right)∥}^{2} \end{array}$$

is minimized by an optimal statistician that can use the posterior mean as an estimator, yielding the *minimum mean square error* (MMSE)

(18) $$\begin{array}{c} \mathrm{MMSE}=E∥\xi -E_{\xi |Y}{\xi ∥}^{2}\;. \end{array}$$

In the following, we shall denote averages with respect to the posterior as ${\langle \cdot \rangle }_{Y}$.

Another important consequence of this setting is the so-called *Nishimori identities*, which can be stated as follows. Given any continuous bounded function *f* of the data Y, the ground truth ξ and n−1 i.i.d. samples from the posterior ${\left(x^{\left(k\right)}\right)}_{k=2}^{n}$, one has

(19) $$\begin{array}{c} E{\langle f\left(Y,\xi ,{\left(x^{\left(k\right)}\right)}_{k=2}^{n}\right)\rangle }_{Y}=E{\langle f\left(Y,x^{\left(1\right)},{\left(x^{\left(k\right)}\right)}_{k=2}^{n}\right)\rangle }_{Y},, \end{array}$$

where $x^{\left(1\right)}∼P_{\xi |Y}$. An elementary proof can be found in [42]. These identities are enforcing a symmetry between replicas drawn from the posterior and the ground truth. For instance, a direct application of the Nishimori identities yields

(20) $$\begin{array}{c} {\mathrm{MMSE}=E∥\xi -\langle x\rangle }_{Y}{∥}^{2}=E\langle ∥x-{\langle x\rangle }_{Y}{{∥}^{2}\rangle }_{Y}\;. \end{array}$$

It is important to stress that, as it can be seen from the above equation, an optimal statistician is actually able to compute the minimum mean square error using their posterior.

At this point, the reader will have noticed a similarity with the Statistical Mechanics formalism. In fact, it is possible to interpret Z(y) as the partition function of a model with Hamiltonian $-\log p_{Y|\xi =x}\left(y\right)$ and unit inverse absolute temperature. The pressure per particle of such a model would thus be

(21) $$\begin{array}{c} p_{N}\left(y\right)=\frac{1}{N}\log Z\left(y\right)\;,\;{\bar{p}}_{N}=E_{Y}p_{N}\left(Y\right)=-\frac{1}{N}H\left(Y\right)\;, \end{array}$$

namely minus the Shannon entropy of the data per signal component, which is related to the mutual information

(22) $$\begin{array}{c} \frac{1}{N}I_{N}\left(Y;\xi \right)=\frac{1}{N}H\left(Y\right)-\frac{1}{N}H\left(Y|\xi \right)\;. \end{array}$$

The contribution coming from the conditional entropy H(Y∣ξ) can be regarded as due only to the noise, since for fixed ξ, the only randomness in Y is due to Z.

We stress here that Bayes optimality, and the Nishimori identities, under rather mild hypotheses [43] are enough to grant *replica symmetry* in the model, i.e., concentration of the order parameters in the model. For the models we are interested in, the latter can be shown to imply finite-dimensional variational principles for the limiting mutual information.

### 3.2. The Spiked Wigner Model

The spiked Wigner model (WSM) was first introduced in [44] as a model for Principal Component Analysis (PCA), and since then, it was widely studied in recent literature. Without pretension of being exhaustive, we refer the interested reader to [42,45,46,47,48,49,50,51]. For our purposes, we restrict ourselves to the case where the signal is an *N*-dimensional vector of ±1 s, drawn from a Rademacher distribution $\xi_{i}\overset{\mathrm{iid}}{∼}P_{\xi }=\left(\delta_{-1}+\delta_{1}\right)/2$. The function F(;z) is a *Gaussian channel*, namely

(23) $$\begin{array}{c} Y_{ij}=\sqrt{\frac{\mu }{2N}}\xi_{i}\xi_{j}+z_{ij} \end{array}$$

where $z_{ij}\overset{\mathrm{iid}}{∼}N\left(0,1\right)$, and μ is a positive parameter called the *signal-to-noise* ratio. The statistician is tasked with the recovery of ξ given the observations Y. The Bayes-optimal posterior measure for this inference problem can be written directly as a Boltzmann–Gibbs random measure thanks to the Gaussian nature of the likelihood:

(24) $$\begin{array}{c} G_{N}\left(\sigma \right)=\frac{1}{Z(z,\xi )}e^{-H_{N}\left(\sigma ;z,\xi \right)}\;,\;-H_{N}\left(\sigma ;z,\xi \right)=\sum_{i,j=1}^{N}\sqrt{\frac{\mu }{2N}}z_{ij}\sigma_{i}\sigma_{j}+\frac{\mu }{2N}\sigma_{i}\xi_{i}\sigma_{j}\xi_{j} \end{array}$$

where we have already exploited the fact that ${\left(\xi_{i}\right)}^{2}=\sigma_{i}^{2}=1$. We are denoting the posterior samples with σ. Since the quantity we are interested in is the quenched pressure of this model

(25) $$\begin{array}{c} {\bar{p}}_{N}=\frac{1}{N}E\log\sum_{\sigma \in {\left\{-1,1\right\}}^{N}}\exp\left[-H_{N}\left(\sigma ;z,\xi \right)\right] \end{array}$$

that is connected to the mutual information $I_{N}\left(Y;\xi \right)/N$ by a simple shift with an additive constant, we are allowed to perform a gauge transformation without altering its value:

(26) $$\begin{array}{c} z_{ij}\mapsto z_{ij}\xi_{i}\xi_{j}\;,\;\sigma_{i}\sigma_{j}\mapsto \sigma_{i}\sigma_{j}\xi_{i}\xi_{j}\;. \end{array}$$

This results in a Hamiltonian that is now independent of the original ground-truth signal

(27) $$\begin{array}{c} -H_{N}^{\prime }\left(\sigma ;z\right)=\sum_{i,j=1}^{N}\left(\sqrt{\frac{\mu }{2N}}z_{ij}+\frac{\mu }{2N}\right)\sigma_{i}\sigma_{j} \end{array}$$

and the coupling between spins are Gaussian random variables with a mean equal to their variance. This condition identifies a peculiar region of the phase space of a spin-glass model, which is called *Nishimori line*. In fact, the Nishimori identities were first discovered and studied in the context of gauge spin-glasses. Despite looking simpler, the above model retains most of the features we need for our study.

For inference models with additive Gaussian noise, like the one above, it is possible to prove the so-called *I-MMSE* relation:

(28) $$\begin{array}{c} \frac{d}{d\mu }\frac{I_{N}\left(Y;\xi \right)}{N}=\frac{1}{4N^{2}}E{∥\xi \xi^{⊺}-\langle \sigma \sigma^{⊺}\rangle ∥}_{F}^{2}\;, \end{array}$$

where ${∥\cdot ∥}_{F}$ is the Frobenius norm and 〈·〉 denotes the expectation with respect to the Boltzmann–Gibbs measure induced by (27). Hence, once the mutual information is known, the MMSE can be accessed through a derivative with respect to the signal-to-noise ratio. A clarification is in order here: the above is the MMSE on the reconstruction of the rank-one matrix $\xi \xi^{⊺}$, because, due to flip symmetry, here we do not have any actual information on the single vector ξ, but only on the *spike* $\xi \xi^{⊺}$.

### 3.3. Sub-Optimality and Replica Symmetry Breaking

There are several ways to break Bayes optimality. Some examples are that the statistician does not know the signal-to-noise ratio μ [13,52]; the statistician adopts a likelihood different from that of the true model [14]; the statistician adopts a wrong prior [12,53]; combinations of the previous and many others. We will focus on the mismatching priors case, where the statistician not only adopts a wrong prior on the ground-truth elements, but they are not aware of the rank of the spiked matrix hidden inside the noise, which is denoted by *M*. The rest is assumed to be known. The channel of the inference problem is

(29) $$\begin{array}{c} Y_{ij}=\sqrt{\frac{\mu }{2N}}\sum_{k=1}^{M}\xi_{i}^{\left(k\right)}\xi_{j}^{\left(k\right)}+z_{ij}\;. \end{array}$$

If the statistician assumes a Rademacher prior to the signal components and a rank-one hidden matrix, they will write a posterior in the form

(30) $$\begin{array}{c} {/\;P}_{\xi |z,\xi }\left(\sigma \right)=\frac{1}{/\;Z(z,\xi )}\exp\left(-H_{N}\left(\sigma ;z,\xi \right)\right) \end{array}$$

where

(31) $$\begin{array}{c} -H_{N}\left(\sigma ;z,\xi \right)=\sum_{i,j=1}^{N}\left(\sqrt{\frac{\mu }{2N}}z_{ij}+\frac{\mu }{2N}\sum_{k=1}^{P}\xi_{i}^{\left(k\right)}\xi_{j}^{\left(k\right)}\right)\sigma_{i}\sigma_{j} \end{array}$$

The slash on quantities emphasizes that they are not the Bayes-optimal ones. In this setting, one can no longer rely on the Nishimori identities, and in principle, replica symmetry is no longer guaranteed. On the contrary, as we shall argue later on, a mismatch in the prior only is already sufficient to cause *replica symmetry breaking*.

## 4. The Model

Let *M* be a fixed integer and k∈{1,…,M}. Consider two independent random collections ${\left(z_{ij}\right)}_{i,j\leq N}\overset{\mathrm{iid}}{∼}N\left(0,1\right)$ and ${\left(\xi_{i}^{\left(k\right)}\right)}_{i\leq N}^{k\leq M}\overset{\mathrm{iid}}{∼}P_{\xi }$ where $P_{\xi }$ is such that $E[\xi^{4}]<\infty$. The above random collections play the role of quenched disorder in the model. Consider *N* Ising spins $\sigma ={\left(\sigma_{i}\right)}_{i\leq N}\in {\left\{+1,-1\right\}}^{N}$ and the Hamiltonian function

(32) $$\begin{array}{c} \begin{array}{cc} H_{N}\left(\sigma ;\mu ,\nu ,\lambda \right) & \equiv H_{N}\left(\sigma \right)=H_{N}^{int}\left(\sigma \right)-h\cdot \sigma \\ \; & =-\sum_{i,j=1}^{N}\left(\sqrt{\frac{\mu }{2N}}z_{ij}+\frac{\nu }{2N}\sum_{k\leq M}\xi_{i}^{\left(k\right)}\xi_{j}^{\left(k\right)}\right)\sigma_{i}\sigma_{j}-\sum_{k\leq M}\lambda_{k}\sum_{i=1}^{N}\xi_{i}^{\left(k\right)}\sigma_{i} \end{array} \end{array}$$

with $\mu ,\nu \geq 0,\;\lambda ={\left(\lambda_{k}\right)}_{k\leq M}\in R^{M}$. Here, $H_{N}^{int}$ is the interacting part while

(33) $$h={\left(h_{i}\right)}_{i\leq N}\equiv h\left(\lambda ,\xi \right)={\left(\sum_{k\leq M}\lambda_{k}\xi_{i}^{\left(k\right)}\right)}_{i\leq N}$$

denotes the random external field acting on the spins. The Hamiltonian (32) is determined by the choice of M,μ,ν,λ and $P_{\xi }$. For μ=ν, the interaction term $H_{N}^{int}$ coincides with the Hamiltonian (31). Note that for some special choices of the parameters, we recover some well-known spin glass models:

- ν=0 gives the SK model (1) at $\beta =\sqrt{\mu }$ and random external field h.
- μ=0 gives the Hopfield model [6,7,18] with a finite number of patterns ${\left(\xi^{\left(k\right)}\right)}^{k\leq M}$.
- $M=1,\nu =\mu ,\lambda_{1}=0$ and $P_{\xi }=\frac{1}{2}\left(\delta_{-1}+\delta_{-1}\right)$ gives the SK model on the Nishimori line (27). As we have seen in Section 3, the latter can be also viewed as a spiked Wigner model in the Bayesian-optimal setting.

Notice that the entire model can be interpreted as a *Hopfield model* where the traditional Hebbian matrix $\sum_{k\leq M}\xi_{i}^{\left(k\right)}\xi_{j}^{\left(k\right)}$ is corrupted by Gaussian noise. Furthermore, if the Hebbian coupling is replaced by a constant matrix, the model reduces to an SK model with the addition of a ferromagnetic interaction, and it was studied in [54].

Our main result is the computation of the thermodynamic limit of the pressure per particle

(34) $$\begin{array}{c} p_{N}\left(\mu ,\nu ,\lambda \right)=\frac{1}{N}\log\sum_{\sigma \in {\left\{-1,1\right\}}^{N}}e^{-H_{N}\left(\sigma \right)} \end{array}$$

whose variance can be shown to converge to 0 as an $O(N^{-1})$, namely:

**Lemma** **1.** *Assume $E\xi_{1}^{4}<\infty$. Then, for any μ,ν∈R and $\lambda \in R^{K}$*

(35) $$\begin{array}{c} E{\left[p_{N}\left(\mu ,\nu ,\lambda \right)-Ep_{N}\left(\mu ,\nu ,\lambda \right)\right]}^{2}\leq \frac{K}{N} \end{array}$$

*where K is a suitable positive constant.*

We thus focus on ${\bar{p}}_{N}\left(\mu ,\nu ,\lambda \right)=Ep_{N}\left(\mu ,\nu ,\lambda \right)$. The proof of this lemma makes use of the Efron–Stein concentration inequality to bound the variance, and it is simple but tedious. It follows closely that of ([12], Lemma 9). We are now in a position to state our main theorem:

**Theorem** **2** (Variational solution). *If $E[\xi^{4}]<+\infty$ then*

(36) $$\begin{array}{c} \lim_{N\to \infty }{\bar{p}}_{N}\left(\mu ,\nu ,\lambda \right)=\underset{x\in R^{M}}{\sup}\varphi \left(x;\mu ,\nu ,\lambda \right) \end{array}$$

*where*

(37) $$\begin{array}{c} \varphi \left(x;\mu ,\nu ,\lambda \right):=-\frac{{\nu |x|}^{2}}{2}+E\;P\left(\sqrt{\mu },h\left(x,\lambda ,\xi \right)\right)\;,\;\;x={\left(x_{k}\right)}_{k\leq M}\in R^{M} \end{array}$$

*and P is the Parisi functional (5) with a random external field*

(38) $$\begin{array}{c} h\left(x,\lambda ,\xi \right)=\sum_{k\leq M}\left(\lambda_{k}+\nu x_{k}\right)\;\xi^{\left(k\right)} \end{array}$$

*and E denotes the expectation with respect to $\xi^{\left(k\right)}\overset{\mathrm{iid}}{∼}P_{\xi }$. The consistency equations are*

(39) $$x_{k}\;=\;\frac{\partial }{\partial x_{k}}\;E\;P\left(\sqrt{\mu },h\left(x,\lambda ,\xi \right)\right),\;\;k\leq M$$

*Moreover, there exists C>0 such that for any k≤M, one has $|x_{k}|\leq C$ and the supremum in (36) can be restricted to ${\left[-C,C\right]}^{M}$.*

The proof of the theorem is based on the concentration of the Mattis magnetization, which is the normalized scalar product between a spin-configuration (or sample from the wrong posterior measure) and one of the $\xi^{\left(k\right)}$:

(40) $$m_{N}\left(\sigma |\xi^{\left(k\right)}\right)=\frac{1}{N}\sum_{i=1}^{N}\sigma_{i}\;\xi_{i}^{\left(k\right)}\;=\frac{1}{N}\sigma \cdot \xi^{\left(k\right)}\;.$$

The Hamiltonian can thus be rewritten using (40) in the following form:

(41) $$\begin{array}{c} H_{N}\left(\sigma \right)=\sqrt{\mu }\;H_{N}^{SK}\left(\sigma \right)-N\;\sum_{k\leq M}\left[\frac{\nu }{2}m_{N}^{2}\left(\sigma |\xi^{\left(k\right)}\right)+\lambda_{k}m_{N}\left(\sigma |\xi^{\left(k\right)}\right)\right] \end{array}$$

The Mattis magnetization, in fact, plays the role of an order parameter for this model. The concentration we can prove is only an integral average over some suitably small magnetic fields, which is still sufficient for our purposes:

**Proposition** **1** (Concentration of Mattis Magnetizations). *Consider a k such that k≤M. Let $\epsilon_{k}\in \left[s_{N},2s_{N}\right]$ with $s_{N}=\frac{1}{2}N^{-\alpha }$, α∈(0,1/(2M)) for all k≤M. For any y∈R, we denote by ${\langle \cdot \rangle }_{N,y}$ the Boltzmann–Gibbs measure induced by the Hamiltonian $H_{N,y}\left(\sigma \right)=H_{N}\left(\sigma \right)-y\;\sigma \cdot \xi^{\left(k\right)}$. Then*

(42) $$\begin{array}{c} \lim_{N\to \infty }\frac{1}{s_{N}^{M}}\int_{s_{N}}^{2s_{N}}\prod_{\ell \leq M}d\epsilon_{\ell }\;E{\langle {\left(m_{N}\left(\sigma |\xi^{\left(k\right)}\right)-E{\langle m_{N}\left(\sigma |\xi^{\left(k\right)}\right)\rangle }_{N,\epsilon }\right)}^{2}\rangle }_{N,\epsilon }=0\;, \end{array}$$

*for all μ,ν≥0 and $\lambda \in R^{M}$.*

We shall omit the proof of the above result as it is completely analogous to the one in [12]. We will need an intermediate lemma that leads to it (see Lemma 2 later) together with a second key ingredient: the *adaptive interpolation* technique [48] combined with Guerra’s replica symmetry-breaking upper bound for the quenched pressure of the SK model [8].

**Proof** **of** **Theorem** **2**. Here, we outline the main steps of the proof of the variational principle for the thermodynamic limit. The proof is achieved via two bounds that match in the N→∞ limit. Let us start by defining the interpolating Hamiltonian

(43) $$\begin{array}{c} \begin{array}{cc} H_{N}\left(t;\sigma \right): & =H_{N}\left(\sigma ;\mu ,\left(1-t\right)\nu ,\lambda +R_{\epsilon }\left(t\right)\right)=\\ \; & =\sqrt{\mu }H_{N}^{SK}\left(\sigma \right)-\left(1-t\right)\frac{N\nu }{2}\sum_{k\leq M}m_{N}^{2}\left(\sigma |\xi^{\left(k\right)}\right)-N\sum_{k\leq M}\left(\lambda_{k}+R_{\epsilon }^{\left(k\right)}\left(t\right)\right)m_{N}\left(\sigma |\xi^{\left(k\right)}\right) \end{array} \end{array}$$

where $R_{\epsilon }={\left(R_{\epsilon }^{\left(k\right)}\right)}_{k\leq M}$ and

(44) $$\begin{array}{c} R_{\epsilon }^{\left(k\right)}\left(t\right)=\epsilon_{k}+\nu \int_{0}^{t}ds\;r_{\epsilon }^{\left(k\right)}\left(s\right)\;,\;\epsilon_{k}\in \left[s_{N},2s_{N}\right]\;,\;s_{N}=\frac{N^{-\alpha }}{2} \end{array}$$

with α∈(0,1/(2M)) and where the interpolating functions $r_{\epsilon }^{\left(k\right)}$, that must be continuously differentiable in [0,1] and non negative, will be suitably chosen. With this interpolation, one is able to prove the following sum rule:

**Proposition** **2.** *The following sum rule holds:*

(45) $$\begin{array}{c} {\bar{p}}_{N}\left(\mu ,\nu ,\lambda \right)={\bar{p}}_{N}^{SK}\left(\sqrt{\mu },\lambda +R_{\epsilon }\left(1\right)\right)-\frac{\nu }{2}\int_{0}^{1}dt\sum_{k\leq M}\left[r_{\epsilon }^{(k)2}\left(t\right)-\Delta_{\epsilon }^{\left(k\right)}\left(t\right)\right]+O\left(s_{N}\right) \end{array}$$

*where*

(46) $$\begin{array}{c} \Delta_{\epsilon }^{\left(k\right)}\left(t\right):=E{\langle {\left(m_{N}\left(\sigma |\xi^{\left(k\right)}\right)-r_{\epsilon }^{\left(k\right)}\left(t\right)\right)}^{2}\rangle }_{N,R_{\epsilon }\left(t\right)}\;. \end{array}$$

The proof consists of the computation of the derivative of the interpolating pressure related to the model (43). It follows closely that of ([12], Proposition 7), to which we refer the interested reader. Since the remainder $\Delta_{\epsilon }^{\left(k\right)}$ is non-negative, the above proposition already yields a bound for the quenched pressure of our model when we choose $r_{\epsilon }^{\left(k\right)}=x_{k}\in R$ constant:

(47) $$\begin{array}{c} \underset{N\to \infty }{lim inf}{\bar{p}}_{N}\left(\mu ,\nu ,\lambda \right)\geq \underset{x\in R^{M}}{\sup}\varphi \left(x;\mu ,\nu ,\lambda \right) \end{array}$$

where we used Lipschitz continuity of the SK pressure in the magnetic fields.

The upper bound requires more attention. First, we notice that ${\bar{p}}_{N}^{SK}\left(\sqrt{\mu },\lambda +R_{\epsilon }\left(1\right)\right)$ is convex in the magnetic fields and that $R_{\epsilon }^{\left(k\right)}\left(1\right)=\int_{0}^{1}dt\left(\epsilon_{k}+\nu r_{\epsilon }^{\left(k\right)}\left(t\right)\right)$. Hence, we can use Jensen’s inequality and Lipschitz continuity of ${\bar{p}}^{SK}$ to obtain:

(48) $$\begin{array}{c} {\bar{p}}_{N}\left(\mu ,\nu ,\lambda \right)\leq \int_{0}^{1}dt\left[{\bar{p}}_{N}^{SK}\left(\sqrt{\mu },\lambda +r_{\epsilon }\left(t\right)\right)-\nu \sum_{k_{\leq }M}\frac{r_{\epsilon }^{(k)2}\left(t\right)}{2}\right]+\frac{\nu }{2}\sum_{k\leq M}\int_{0}^{1}\Delta_{\epsilon }^{\left(k\right)}\left(t\right)\;dt+O\left(s_{N}\right)\;. \end{array}$$

Now, we use Guerra’s bound for the SK pressure, that, importantly, is uniform in *N*, and we average over ϵ on both sides

(49) $$\begin{array}{c} {\bar{p}}_{N}\left(\mu ,\nu ,\lambda \right)\leq E_{\epsilon }\int_{0}^{1}\varphi \left(r_{\epsilon }\left(t\right);\mu ,\nu ,\lambda \right)\;dt+\frac{\nu }{2}\sum_{k\leq M}E_{\epsilon }\int_{0}^{1}\Delta_{\epsilon }^{\left(k\right)}\left(t\right)\;dt\;+O\left(s_{N}\right)\leq \\ \leq \underset{x\in R^{M}}{\sup}\varphi \left(x;\mu ,\nu ,\lambda \right)+\frac{\nu }{2}\sum_{k\leq M}E_{\epsilon }\int_{0}^{1}\Delta_{\epsilon }^{\left(k\right)}\left(t\right)\;dt+O\left(s_{N}\right)\;. \end{array}$$

What remains to do is to prove that $E_{\epsilon }\Delta_{\epsilon }^{\left(k\right)}\left(t\right)\overset{N\to \infty }{\to }0$ for a proper choice of the interpolating functions $R_{\epsilon }$. The choice is made through a system of coupled ODEs

(50) $$\begin{array}{c} {\dot{R}}_{\epsilon }^{\left(k\right)}\left(t\right)\equiv \nu r_{\epsilon }^{\left(k\right)}\left(t\right)=\nu E{\langle m_{N}\left(\sigma |\xi^{\left(k\right)}\right)\rangle }_{N,R_{\epsilon }\left(t\right)}\;,\;R_{\epsilon }^{\left(k\right)}\left(0\right)=\epsilon_{k}\;,\;\forall k\leq M\;. \end{array}$$

One can easily check that the above system is regular enough to admit a unique solution on the interval t∈[0,1]. In this case, the remainder to push to 0 would appear as

(51) $$\begin{array}{c} \frac{1}{s_{N}^{M}}\int_{s_{N}}^{2s_{N}}\prod_{\ell \leq M}d\epsilon_{\ell }\;E{\langle {\left(m_{N}\left(\sigma |\xi^{\left(k\right)}\right)-E{\langle m_{N}\left(\sigma |\xi^{\left(k\right)}\right)\rangle }_{N,R_{\epsilon }\left(t\right)}\right)}^{2}\rangle }_{N,R_{\epsilon }\left(t\right)}\;. \end{array}$$

The goal is now to apply a concentration lemma here:

**Lemma** **2.** *Let $y\in \left[y_{1},y_{2}\right],\;\delta \in \left(0,1\right)$ and denote by ${\langle \cdot \rangle }_{N,y}$ the Boltzmann–Gibbs expectation associated to the Hamiltonian $H_{N}\left(\sigma ;\mu ,\nu ,\lambda +ye_{k}\right)$ where k≤M and $e_{k}$ is the k-th canonical basis vector of $R^{M}$. Then*

(52) $$\begin{array}{cc} \; & E{\langle {\left(m_{N}\left(\sigma |\xi^{\left(k\right)}\right)-{\langle m_{N}\left(\sigma |\xi^{\left(k\right)}\right)\rangle }_{N,y}\right)}^{2}\rangle }_{N,y}=\frac{1}{N}\frac{d^{2}}{dy^{2}}{\bar{p}}_{N}\left(\mu ,\nu ,\lambda +ye_{k}\right) \end{array}$$

(53) $$\begin{array}{c} \begin{array}{cc} \; & E\left[{\left({\langle m_{N}\left(\sigma |\xi \right)\rangle }_{N,y}-E{\langle m_{N}\left(\sigma |\xi \right)\rangle }_{N,y}\right)}^{2}\right]\leq \frac{12K}{\delta^{2}N}+\\ & +8\sqrt{a}\frac{d}{dy}\left[{\bar{p}}_{N}\left(\mu ,\nu ,\lambda +\left(y+\delta \right)e_{k}\right)-{\bar{p}}_{N}\left(\mu ,\nu ,\lambda +\left(y-\delta \right)e_{k}\right)\right] \end{array} \end{array}$$

*with K a positive constant.*

Notice that the integral in (51) is over ϵ and not over the effective magnetic field of the model, which is instead $R_{\epsilon }\left(t\right)$. Nevertheless, we can integrate over the magnetic fields $R_{\epsilon }\left(t\right)$ with a change of variables. This involves a Jacobian that is larger than 1. In fact, thanks to Liouville’s theorem ([55], Corollary 3.1, Chapter V), one can prove that

(54) $$\begin{array}{c} \det\frac{\partial R_{\epsilon }\left(t\right)}{\partial \epsilon }=\exp\left[\int_{0}^{t}\nu \sum_{k\leq M}\frac{\partial }{\partial R_{\epsilon }^{\left(k\right)}\left(s\right)}E{\langle m_{N}\left(\sigma |\xi^{\left(k\right)}\right)\rangle }_{N,R_{\epsilon }\left(s\right)}\;ds\right]=\\ \exp\left[\int_{0}^{t}N\nu \sum_{k\leq M}E{\langle {\left(m_{N}\left(\sigma |\xi^{\left(k\right)}\right)-E{\langle m_{N}\left(\sigma |\xi^{\left(k\right)}\right)\rangle }_{N,R_{\epsilon }\left(s\right)}\right)}^{2}\rangle }_{N,R_{\epsilon }\left(s\right)}\;ds\right]\geq 1\;, \end{array}$$

when ν≥0.

This allows us to bound the thermal fluctuations in (51) using (52) and then Liouville’s theorem:

(55) $$\begin{array}{c} \Delta_{T}^{\left(k\right)}:=\frac{1}{s_{N}^{M}}\int_{s_{N}}^{2s_{N}}\prod_{\ell \leq M}d\epsilon_{\ell }\;E{\langle {\left(m_{N}\left(\sigma |\xi^{\left(k\right)}\right)-{\langle m_{N}\left(\sigma |\xi^{\left(k\right)}\right)\rangle }_{N,R_{\epsilon }\left(t\right)}\right)}^{2}\rangle }_{N,R_{\epsilon }\left(t\right)}\leq \\ \leq \frac{1}{s_{N}^{M}}\prod_{\ell \leq M}\int_{R_{s_{N}}^{\left(\ell \right)}\left(t\right)}^{R_{2s_{N}}^{\left(\ell \right)}\left(t\right)}dh_{\ell }\;\frac{1}{N}\frac{d^{2}}{dh_{k}^{2}}{\bar{p}}_{N}\left(\mu ,\nu ,\lambda +h\right)=\\ =\frac{1}{Ns_{N}^{M}}\prod_{\ell \leq M,\neq k}\int_{R_{s_{N}}^{\left(\ell \right)}\left(t\right)}^{R_{2s_{N}}^{\left(\ell \right)}\left(t\right)}dh_{\ell }\;\left[E{\langle m_{N}\left(\sigma |\xi^{\left(k\right)}\right)\rangle }_{N,h;h_{k}=R_{2s_{N}}^{\left(k\right)}}-E{\langle m_{N}\left(\sigma |\xi^{\left(k\right)}\right)\rangle }_{N,h;h_{k}=R_{s_{N}}^{\left(k\right)}}\right] \end{array}$$

Since $\xi_{i}$ has a bounded second moment, using Cauchy–Schwarz inequality, one can show that $|E{\langle m_{N}\left(\sigma |\xi^{\left(k\right)}\right)\rangle }_{N,h}|$ is uniformly bounded by a constant *C*. Hence, $|R_{\epsilon }^{\left(k\right)}\left(t\right)|\leq \epsilon_{k}+Ct\nu \leq 1+C\nu$ for any k≤M by construction (recall (44) and (50)). Therefore, $\Delta_{T}=O\left(\frac{1}{Ns_{N}^{M}}\right)$.

The fluctuations induced by the disorder can be bounded in a very similar fashion using (53):

(56) $$\begin{array}{c} \Delta_{D}^{\left(k\right)}:=\frac{1}{s_{N}^{M}}\int_{s_{N}}^{2s_{N}}\prod_{\ell \leq M}d\epsilon_{\ell }\;E{\left({\langle m_{N}\left(\sigma |\xi^{\left(k\right)}\right)\rangle }_{N,R_{\epsilon }\left(t\right)}-E{\langle m_{N}\left(\sigma |\xi^{\left(k\right)}\right)\rangle }_{N,R_{\epsilon }\left(t\right)}\right)}^{2}=O\left(\frac{1}{N\delta^{2}}+\frac{\delta }{s_{N}^{M}}\right) \end{array}$$

Hence, overall (51), that equals $\Delta_{T}^{\left(k\right)}+\Delta_{D}^{\left(k\right)}$ is a $O\left(\frac{1}{Ns_{N}^{M}}+\frac{1}{N\delta^{2}}+\frac{\delta }{s_{N}^{M}}\right)$. δ can be chosen as a function of *N* in order to optimize the convergence rate: $\delta =s_{N}^{2M/3}N^{-1/3}$. Using Fubini’s theorem in (49) to exchange the *t* and ϵ averages and then Dominated Convergence, one concludes the proof. □

From the the variational problem (36), we can deduce also the differentiability properties of the limiting pressure obtaining the average values of the relevant thermodynamic quantities of the model:

**Corollary** **1.** *Let $p\left(\mu ,\nu ,\lambda \right)=\lim_{N\to \infty }{\bar{p}}_{N}\left(\mu ,\nu ,\lambda \right)$, and $\Omega_{\mu ,\nu ,\lambda }={\mathrm{argmax}}_{x\in {\left[-C,C\right]}^{M}}\varphi \left(x;\mu ,\nu ,\lambda \right)$. Then*

- *If $\Omega_{\mu ,\nu ,\lambda }=\left\{\bar{x}\right\}$ is a singleton with $\bar{x}={\left({\bar{x}}_{k}\right)}_{k\leq M}$ then for any k≤M, one has*
(57) $$\begin{array}{c} \lim_{N\to \infty }E{\langle m_{N}\left(\sigma |\xi^{k}\right)\rangle }_{N}\;=\;\frac{\partial }{\partial \lambda_{k}}p\left(\mu ,\nu ,\lambda \right)\;=\;{\bar{x}}_{k} \end{array}$$
*and*
(58) $$\begin{array}{c} \lim_{N\to \infty }E{\langle q_{12}^{2}\rangle }_{N}=1-4\frac{\partial }{\partial \mu }p\left(\mu ,\nu ,\lambda \right)=\int q^{2}d\chi^{*}\left(q\right)\;. \end{array}$$
*where $\chi^{*}\left(q\right)$ denotes the unique measure solving the Parisi variational principle in Theorem 1.*
- *If $\left\{{\left|x\right|}^{2},x\in \Omega_{\mu ,\nu ,\lambda }\right\}=\left\{\right|\bar{x}{|}^{2}\}$ is a singleton then*
(59) $$\begin{array}{c} \frac{\partial }{\partial \nu }p\left(\mu ,\nu ,\lambda \right)=\frac{|\bar{x}{|}^{2}}{2}\;. \end{array}$$

*More generally, let y be one of the variables $\sqrt{\mu },\nu ,\lambda_{1},\ldots ,\lambda_{M}$, then the function y↦p(μ,ν,λ) is convex. By Danskin theorem (see [56]), y↦p(μ,ν,λ) is differentiable if and only if the set $\left\{\frac{\partial \varphi (x;\mu ,\nu ,\lambda )}{\partial y}\;,\;x\in \Omega_{\mu ,\nu ,\lambda }\right\}$ is a singleton.*

## 5. Conclusions and Perspectives

In this paper, we offer an overview of the Parisi formula from a mathematical physics perspective, emphasizing its potential applications, particularly in addressing the mismatched inference problem outlined earlier. Building upon our previous work [12], we investigate a scenario where a statistician, tasked with reconstructing a finite-rank matrix, lacks knowledge about the underlying matrix generation process, including both the matrix elements and its rank. We consider the case in which the statistician assumes a rank-one matrix, leading to a mismatch between the "true" Bayes posterior and the one used for inference. Our key contribution is the proof that, contrary to what happens in the Bayes-optimal setting, this Bayesian mismatch induces replica symmetry breaking in the model. Consequently, we express the pressure of the corresponding spin glass as an infinite-dimensional variational principle over the space of distributions on [0, 1].

The chosen mismatch scenario shares some similarities with those studied in [57,58] with the fundamental difference being that here the rank of the hidden matrix is finite. In a recent work [59], the authors consider a general case of mismatch, which includes mismatching priors and likelihoods. The mentioned paper proves a universality property with respect to the likelihood assumed by the statistician provided that observations remain independent given the ground truth.

Despite these advancements, all the proofs available so far in the literature break down when considering a high-rank hidden matrix. To rigorously comprehend this scenario, addressing the solution of the Hopfield model is of crucial importance. However, to the best of our knowledge, its complete solution remains elusive [5,6].

## Data Availability

No new data were created or analyzed in this study. Data sharing is not applicable to this article.
